# Autotaxin inhibitor IOA-289 reduces gastrointestinal cancer progression in preclinical models

**DOI:** 10.1186/s13046-023-02780-4

**Published:** 2023-08-08

**Authors:** Matteo Centonze, Giusy Di Conza, Michael Lahn, Isabel Fabregat, Francesco Dituri, Isabella Gigante, Grazia Serino, Rosanna Scialpi, Livianna Carrieri, Roberto Negro, Elena Pizzuto, Gianluigi Giannelli

**Affiliations:** 1 National Institute of Gastroenterology - IRCCS “Saverio de Bellis”, Via Turi 27, 70013 Castellana Grotte, Italy; 2 iOnctura SA, Avenue Secheron 15, 1202 Geneva, Switzerland; 3 TGF-β and Cancer Group, Oncobell Program, Bellvitge Biomedical Research Institute (IDIBELL) and CIBEREHD – ISCIII, Barcelona, Spain

**Keywords:** Autotaxin, Lysophosphatidic acid, Gastrointestinal cancers

## Abstract

**Background:**

Autotaxin (ATX) is a secreted enzyme that converts lysophosphatidylcholine to lysophosphatidic acid (LPA). LPA stimulates cell proliferation and migration and promotes wound repair following tissue damage. ATX levels are directly correlated with stage and grade in several human cancers. Several small molecule ATX inhibitors have been developed in recent years. IOA-289 is a potent ATX inhibitor, developed to treat cancers containing fibrosis. In this study, we tested IOA-289 treatment on different gastrointestinal tract tumor cell lines, in order to evaluate its effects on viability and motility.

**Methods:**

To determine the effects on cell viability and proliferation of treatment with increasing concentrations of IOA-289, we used the crystal violet assay, a clonogenic assay in matrigel, and we evaluated the inhibitor’s effect on formation of 3D spheroids in an in vitro model. The effect of IOA-289 on cell cycle phases was analysed with a redox dye reagent. Cell migration capacity was evaluated by wound healing assay and transwell migration assay. To evaluate the pro-apoptotic effect of the inhibitor, cells were stained with Annexin V and immunofluorescence and flow cytometry analysis were performed. An antibody array was also used, to discriminate, in various samples, the differential expression of 43 proteins involved in the apoptosis pathway.

**Results:**

We found that IOA-289 is able to inhibit both growth and migration of gastrointestinal tract tumor cell lines, both in 2D (crystal violet assay) and 3D in vitro models (spheroid formation and clonogenic assay in matrigel). This effect is dose-dependent, and the drug is most effective when administered in FBS-free culture medium. The inhibitory effect on cell growth is due to a pro-apoptotic effect of IOA-289. Staining with FITC-conjugated Annexin V showed that IOA-289 induced a dose-dependent increase in fluorescence following incubation for 24 h, and apoptotic cells were also distinguished in flow cytometry using Annexin/PI staining. The antibody array shows that treatment with IOA-289 causes the increased expression of several pro-apoptotic proteins in all tested cell lines.

**Conclusions:**

These results indicate that IOA-289 may be an effective drug for the treatment of tumors of the gastrointestinal tract, particularly those characterized by a high degree of fibrosis.

## Introduction

Lysophosphatidic acid (LPA) is a bioactive phospholipid that can act as a signaling molecule, regulating many cellular effects in various cell types. LPA can be synthesized by an intracellular pathway, involving phospholipids or diacylglicerol as precursors, and an extracellular pathway in which LPA is generated from lysophosphatidylcholine, normally present in the extracellular leaflet of plasma membranes or bound to other proteins, such as albumin [1]. Lysophosphatidylcholine is converted to LPA by Autotaxin (ATX), a secreted glycoprotein with lysophospholipase D activity encoded by the ectonucleotide pyrophosphatase phosphodiesterase 2 (*ENPP2*) gene [2].

There are five ATX isoforms distributed in different tissues [3]. This enzyme is composed of two N-terminal somatomedin B (SMB)-like domains, a central phosphodiesterase (PDE) domain containing the active catalytic site, and a C-terminal nuclease (NUC)-like domain. The N-terminal SMB-like domains are cysteine-enriched, and are involved in the interaction of ATX with β3 integrins on the cell surface [4]. The PDE domain also contains a hydrophobic lipid binding pocket that can bind different LPC and LPA species [5, 6]. The binding of ATX with integrins localizes ATX activity on the cell surface, allowing the generation and delivery of LPA in the vicinity of its surface receptors [7, 8]. LPA interacts with six different G protein-coupled receptors (LPAR 1–6) to activate several pathways and downstream signaling molecules such as Rho, Ras PLC, PI3K [9], regulating physiological processes like cell survival, proliferation and motility.

It is well known that ATX and its product LPA play an important role in physiological and pathological conditions. In healthy individuals, ATX is normally expressed in various tissue and can be found in biological fluids [10], but an increased expression has been found in several tumor types. In patients with hepatocellular carcinoma (HCC) an overexpression of ATX has been observed compared to that in hepatitis C and healthy patients [11, 12], and increased levels of ATX and LPA have been observed in lung tissue from patients with idiopathic pulmonary fibrosis and lung cancer [13]. The ATX gene is strongly up-regulated in kidney tumour tissue samples from RCC patients [14].

Given the prominent role of the ATX/LPA axis in promoting cancerogenesis, it may be a promising target for the treatment of cancer and inflammation-related diseases, such as chronic hepatitis and pulmonary fibrosis.

In the last years, several small molecules have been developed to act as ATX inhibitors. These molecules can act by competing with LPC for the hydrophobic pocket and by binding to the active site of the enzyme [15]. IOA-289 is an orally available ATX inhibitor in clinical development by iOnctura for the treatment of solid tumors with a high degree of fibrosis [16]. IOA-289 does not bind to the catalytic zinc region of ATX but binds to both the substrate pocket and the LPA carrier channel blocking both functions of ATX. IOA-289 inhibits plasma LPA18:2 with an IC_50_ of 36 nM, and shows similar results for other LPA species. In vitro studies show that IOA-289 does not only modulate tumor growth, but can also modulate the immunosuppressive fibrotic microenvironment [16]. IOA-289 inhibits IL-6 and PAI-1 secretion by activated fibroblasts, and with the BioMAP phenotypic screen it has been shown that IOA-289 can inhibit the expression of fibrosis factors (sIL-6, MCP-1, αSMA, collagen-III) [16]. Moreover,it has been observed in vitro that IOA-289 can inhibit the ability of soluble factors, released by cancer-associated fibroblasts, to stimulate the growth of cancer cells and affect the recruitment of T cells to the tumor site [17, 18].

Aim of this study is to evaluate the effectiveness in experimental preclinical models of the ATX inhibitor IOA-289 on tumor progression of different gastrointestinal malignancies including intrahepatic cholangiocarcinoma (iCCA), HCC, colorectal adenocarcinoma (CRC) and pancreatic cancer (PC).

## Methods

### Cell culture and reagents

Human colorectal adenocarcinoma HT-29 and Caco-2 cells, hepatocellular carcinoma HLE cells, cholangiocarcinoma RBE and KKU-M213 cells and pancreatic carcinoma PANC-1 and MIA PaCa-2 cells were purchased from the American Tissue Culture Collection (*ATCC*, *Manassas, VA*, *USA*). The hepatocellular carcinoma cell line HLF was purchased from JCRB Cell Bank (Japan). All cell lines were cultured in Dulbecco’s Modified Eagle Medium (DMEM) supplemented with 10% foetal bovine serum (FBS), 1 mM pyruvate, 25 mM HEPES, 100 U/mL penicillin–streptomycin (all from Thermo Fisher Scientific, Waltham, MA, USA), and maintained in a humidified atmosphere at 37 °C containing 5% CO_2_. The medium was changed every three days. ATX inhibitor IOA-289 was a kind gift from iOnctura.

### Crystal violet assay

Cells were seeded in 96-well plates at a density of 2000 cells/well in 10% FBS supplemented medium. After 24 h, medium was removed, the cells were washed with PBS and cultured in the presence or absence of FBS and of different concentrations of IOA-289. After the time set for the experiment (24, 48 and 72 h), the cells were fixed with a 4% PFA solution and stained with 0.02% crystal violet for 10 min. Subsequently the plates were washed with water to remove excess dye. The cell-bound dye was redissolved in 1% SDS and the optical density was measured at λ = 570 with a iMark™ Microplate Absorbance Reader (Bio-rad).

### 3D spheroids culture

Spheroids were created using the hanging drop method. For all the experiments, cells were suspended, at a concentration of 50,000 cells/ml, in medium with 0,24% of methylcellulose (Sigma) and supplemented with 2% FBS, in the absence or presence of increasing concentration of IOA-289. Twenty-five microliter drops were pipetted onto the lid of 100 mm dishes, that were inverted over dishes containing 5 ml of cell culture medium to avoid drying. After 3 days of incubation at 37 °C and 10% CO_2_, spheroids were transferred by pipetting onto a low-attachment 6-well culture plate (Corning). Images of 10–20 spheroids per experiment were acquired with a NIKON Eclipse Ti2 microscope and spheroids size was measured with ImageJ software (National Institute of Health, USA).

### Clonogenic assay

The culture surface of a 96-well plate was coated with a thin layer of Matrigel (Corning) (30 µl/well) and incubated for 20 min at 37 °C to allow it to gel. Cells were trypsinized and filtered through a 40 µm mesh to make a single cells suspension. Two microliters of medium containing 2000 cells were mixed with 28 µl of matrigel and layered on top of the previously coated wells. After gelification for 20 min at 37 °C, 90 µl of 10% FBS complete medium were added to the wells. After 24 h, the 10% FBS medium was removed and replaced by 2% FBS medium with 3,9 and 12 µM IOA-289. Medium was changed every 2–3 days. Colonies had been generated after 10–14 days of culture and cell clusters (with at least 50 cells) were counted microscopically without staining using a NIKON Eclipse Ti2 microscope.

### Cell cycle analysis

Cell cycle analysis was performed using a Cell-Clock Assay Kit (Biocolor Life Science Assays, County Antrim, UK.) on cell lines treated with 3,9 and 12 µM IOA-289 for 24 h. Briefly, 2.0 × 10^5^ cells/well were seeded on 24-well plates and incubated at 370C, 5% C02, to > 80% confluence. The cells were then treated with fresh medium containing IOA-289. After 24 h, the cell cycle phases were visualized using the kit according to the manufacturer’s instructions. Images were analysed by ImageJ software to determine the ratio of cells in each cell cycle phase.

### Wound healing assay

Cells were seeded in a 12-well plate in 10% FBS medium and grown to reach a 80–90% confluence. Then, a scratch was made on the cell monolayer using a p200 pipette tip. The plate was washed with sterile PBS to remove the debris and markings were created on the outer bottom of the plate with a tip marker, to be used as reference points. Each cell line was incubated with medium without FBS, containing increasing concentrations of IOA-289 (1–3-9–12 µM). Images were acquired at 0, 24 and 48 h using a confocal microscope Nikon Eclipse Ti2 in bright field microscopy and Image-J software was used to measure the scratched area. Cell migratory ability for wound-healing was assessed by applying the following formula: [(wound area at 0 h) – (wound area at indicated time)] / (wound area at 0 h).

### Transwell migration assay

Cell culture inserts with 8 µM pores (Corning, inc.) were coated with 50 µl of a 10 mg/ml Collagen type 1 solution (Gibco, USA). 1.5 × 10^4^ cells were plated in the upper chamber in serum-free medium, in the absence or presence of 12 µM IOA-289, while the bottom wells were filled with complete medium. The cells were allowed to migrate across the membrane for at least 16 h. After incubation, the cells were fixed with PFA 4% for 10’ and stained with 0.02% crystal violet for 10’. Excess dye was washed off with tap water and the non-migrated cells were scraped off from the upper surface of the membrane with a cotton swab. Cell migratory ability was assessed by counting the average number of cells in at least 5 fields at 10X magnification.

### Annexin V staining

To detect early apoptosis by immunofluorescence, the Annexin V-FITC kit (Miltenyi Biotech, Germany) was used. Cells were seeded and grown on Nunc Lab-Tek II Chamber Slide. After washing with PBS, cells were incubated with IOA-289 3, 9 and 12 µM in serum-free medium. After 2 h cells were washed with Binding Buffer (BB) and stained with a solution of Annexin V-FITC antibody in BB for 15’. After two washes with BB cells were fixed with a 4% paraformaldehyde solution and a coverslip was mounted onto the slide using a DAPI Ready Made Solution with Antifade (Merck, US). Images were acquired using the Nikon Eclipse Ti2 confocal microscope and analyzed with ImageJ software.

### Cytofluorimetric analysis

Flow cytometry analysis of cell apoptosis following treatment with 3, 9 and 12 µM IOA-289 for 24 h was done using the Annexin V-FITC kit, according to the manufacturer’s instructions. Briefly, adherent cells were detached using StemPro Accutase (Thermo Fisher Scientific, Waltham, MA, USA) and mixed with any floating cells; 10^6^ cells were washed with 1 ml of Binding Buffer (BB). The pellet was resuspended with 100 µl of BB, 10 µl of Annexin V-FITC were added and incubated for 15 min in the dark. After another wash with BB, the cell pellet was resuspended with 500 μL of BB, and 5 μL of propidium iodide were added prior to analysis with the Navios flow cytometer, using Kaluza software for quantification and graph plotting (Beckman Coulter).

### Human apoptosis antibody array

The Human Apoptosis Antibody Array Kit (ab134001, Abcam) was used to detect the apoptosis pathway. Briefly, cells were lysed and each membrane was incubated at 4° overnight with 500 µg of total extracted protein. Membranes were then washed and incubated overnight at 4° with Biotin-conjugated Anti-Cytokines. After a incubation of the membranes with HRP-streptavidin for 2 h at room temperature, chemiluminescence was detected using ChemiDoc XRS + (Bio-Rad, USA). The images were analyzed with Image Lab 5.2.1.

### Statistical and bioinformatic analysis

ENPP2 mRNA expression was evaluated in a dataset of CRC, CCA, HCC and PC patients from TGCA using the GEPIA tool (http://gepia2.cancer-pku.cn/) (REF: Tang, Z. et al. (2019) GEPIA2: an enhanced web server for large-scale expression profiling and interactive analysis. Nucleic Acids Res, 10.1093/nar/gkz430.).

Statistical comparison between groups was made with Students’ t-test using GraphPad Prism 6.01 software (GraphPad Software Inc., La Jolla, CA, USA). Statistical significance was set at *p* < 0.05.

## Results

### ENPP2 is expressed in CCA, HCC, CRC and PC patients

**Fig. 1:**
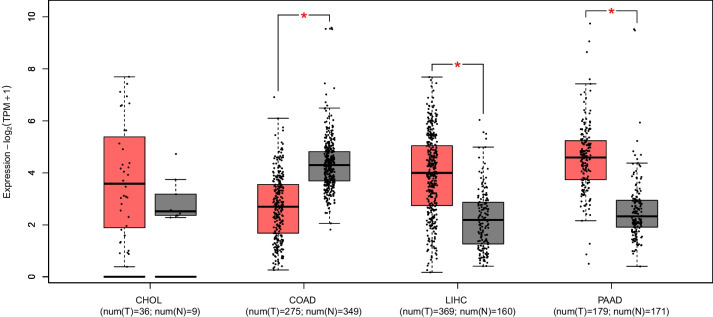
Expression of ENPP2 mRNA evaluated in a dataset of cholangiocarcinoma (CHOL), colorectal cancer (COAD), Hepatocellular carcinoma (LIHC) and pancreatic cancer (PAAD) patients from TGCA using the GEPIA tool. ENPP2 expression in tumor tissue is compared to that in normal peritumoral tissue. ENPP2 expression is upregulated in HCC (*p* < 0.01) and PC (*p* < 0.01), while in CCA the expression is upregulated but not in a statistically significant way* (p* > 0.05),. In CRC, ENPP2 gene expression is downregulated in comparison with normal tissue (*p* < 0.01)

In order to evaluate ENPP2 gene expression levels in patients with cholangiocarcinoma (CCA), hepatocellular carcinoma (HCC), colorectal cancer (CRC), and pancreatic cancer (PC), we used data from TCGA database comparing them with peritumoral counterpart and GTEx database. We found that ENPP2 expression was upregulated in CCA (*p* > 0.05), HCC (*p* < 0.01) and PC (*p* < 0.01). Instead, in CRC, ENPP2 gene expression resulted downregulated in comparison with normal tissue (*p* < 0.01) (Fig. 1).

### IOA-289 affects cell viability and proliferation

**Fig. 2:**
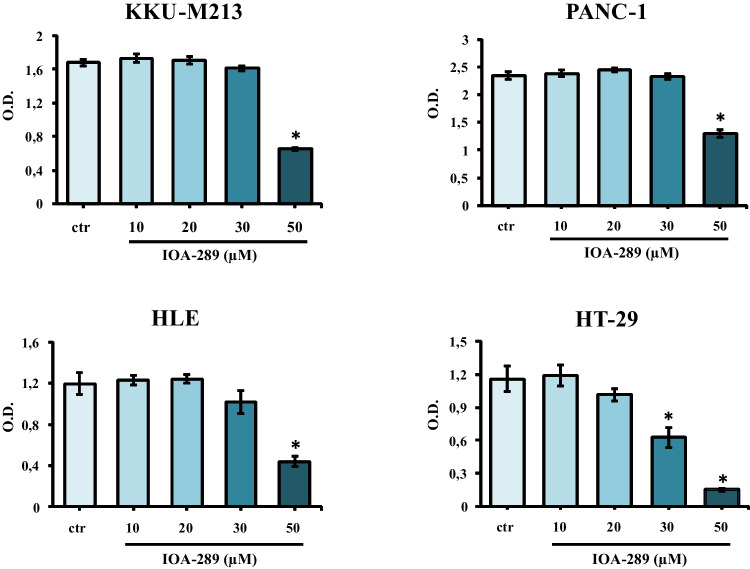
Effect of IOA-289 concentrations ranging from 10 to 50 µM on the viability of KKU-M213, HLE, PANC-1 and HT-29 cells, grown for 72 h in culture medium supplemented with 10% FBS. *P < 0.05

**Fig. 3:**
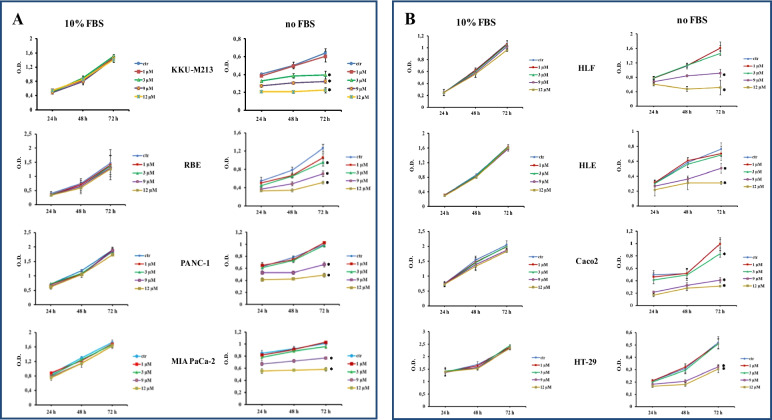
Inhibition by IOA-289 of cell proliferation in gastrointestinal cancer cell lines. Cells were grown in serum free culture medium or in presence of 10% FBS and incubated with increasing IOA-289 concentrations or with DMSO (control cells) for 24, 48 and 72 h. After fixation, proliferation was assessed with the crystal violet staining method. *P < 0.05

**Fig. 4:**
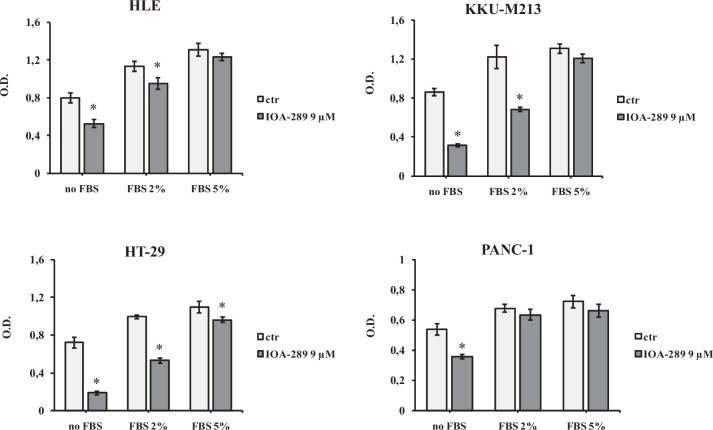
Different responses of KKU-M213, HLE, PANC-1 and HT-29 cells viability to treatment with 9 µM IOA-289 for 72 h in the absence and in the presence of 2 and 5% FBS. *P < 0.05

**Fig. 5:**
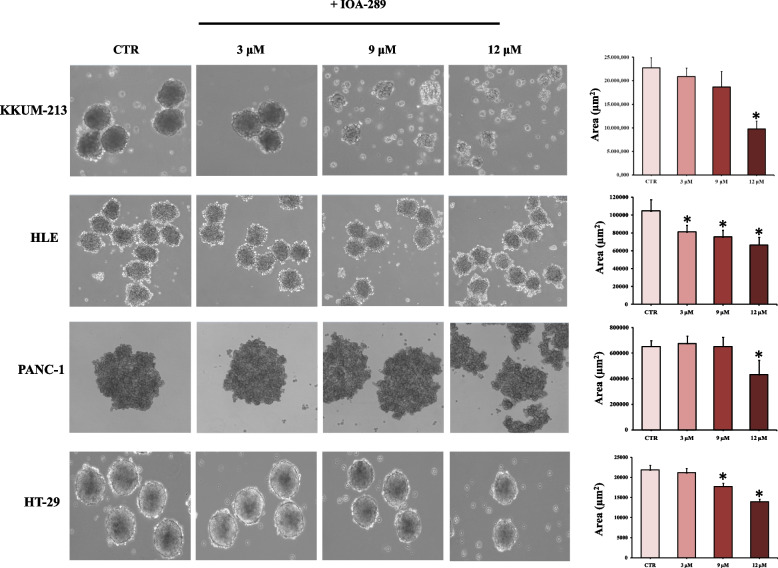
Images of cells incubated in hanging drop method for 72 h, either in the absence or presence of increasing IOA-289 concentratios (3, 9 and 12 µM). Whereupon aggregates size was determined by acquiring the images at 10X magnification and calculating the area of at least 10–20 spheroids for every treatment. Statistical analysis of aggregate size by Student's t-test shows that there is a statistically significant difference in spheroid size between untreated and IOA-289 treated cells (*P < 0.05)

**Fig. 6:**
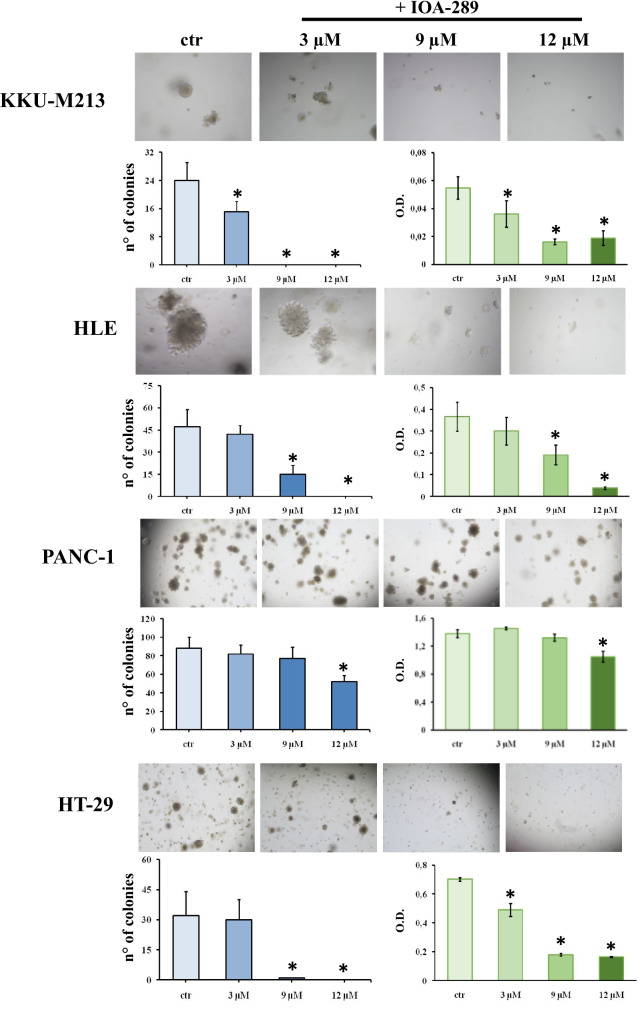
IOA-289 inhibits the formation of colonies from single cells suspension. Cells were seeded with Matrigel and culture for 7–15 days in absence or presence of 3, 9 and 12 µM IOA-289. The total number of colonies with at least 50 cells was counted under a Nikon Ti2 microscopy. Cells treated with IOA-289 form fewer colonies compared to untreated control cells (blue bar graph). In particular, the formation of colonies is completely inhibited treating KKU-M213 and HT-29 cells with 9 and 12 µM IOA-289. The green bar graphs represent the number of viable cells in every treatment, obtained by a colorimetric method. For every experiment *P < 0.05

**Fig. 7:**
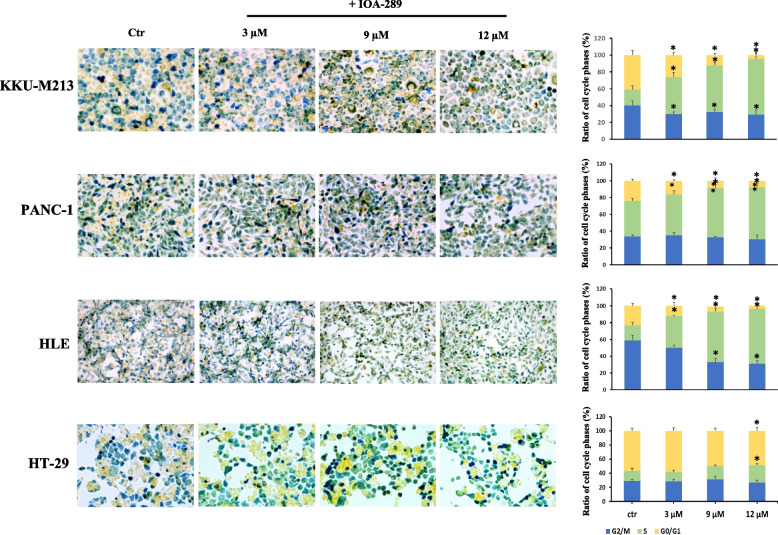
Analysis of changes in cell cycle phases during treatment with IOA-289. Cells from CRC, HCC, PC and CCA were cultured to a > 80% confluence and then treated with 3, 9 and 12 µM IOA-289 for 24 h. The different cell cycle phases were visualized by staining with Biocolor Cell-Clock™ Cell Cycle Assay. Following dye uptake and incubation a distinct color change occurs within cells, with particular color changes being associated with cells in the G0-G1(yellow), S (green), G2 and M Phases (blu). IOA-289 determines an arrest of the cell cycle in S phase, as can be observed particularly for KKU-M213, HLE and PANC-1 cells (*P < 0.05)

To evaluate the anti-proliferative/cytotoxic effects of IOA-289, we treated different cell lines developed from cholangiocarcinoma (CCA), hepatocellular carcinoma (HCC), colorectal cancer (CRC), and pancreatic cancer (PC) with increasing concentration of IOA-289. After the drug treatment, attached cells were fixed and stained with crystal violet dye. This screening method is generally used to evaluate the effect of chemotherapeutics or other anti-cancer drugs on cell survival and proliferation. In our first experiments, IOA-289 was administered to cells for 72 h at concentrations ranging from 10 µM up to 50 µM, in culture medium supplemented with 10% FBS. IOA-289 showed a significant (*p* < 0.05) cytotoxic effect only at the highest concentrations (30 to 50 µM, Fig. 2) in all the cell lines used: KKU-M213,HLE, HT-29, and PANC-1 cells. To test the hypothesis that the presence of serum, and consequently endogenous LPA, could interfere with the IOA-289 activity, we treated eight cancer cell lines with IOA-289 at concentration ranging from 1 µM up to 12 µM, in the presence or absence of 10% FBS. As reported in Fig. 3, IOA-289 displayed a significant (*p* < 0.05) cytotoxic effect in a dose-dependent manner even at lower concentrations (3 to 12 µM). This effect, already evident after 48 h, was strengthened after 72 h of incubation. Thus, IOA-289 showed cytotoxic effect in cells cultured in the absence of FBS but not in those with 10% FBS present in the medium; these effects were consistent for all eight cancer models, and were observed at each repetition (at least three independent experimental repetitions). These results suggest that IOA-289 exerted a strong anti-proliferative effects in all the models and also that FBS interferes with its activity. To further expand our preliminary observations, we challenged HLE, KKU-M213, HT-29 and Panc-1 cells with 9 µM of IOA-289 in the absence or presence of increasing concentrations of FBS. In all the cell models, the presence of FBS significantly (*p* < 0.05) reduced the activity of IOA-289, starting at an FBS concentration of 2%, while the IOA-289 effect was completely abolished at 5% FBS. In Panc-1 cells, FBS blocked drug effectiveness even at the lowest concentration (Fig. 4). In conclusion, IOA-289 selectively inhibits LPA, leading to an anti-proliferative effect, but in presence of FBS the drug effectiveness is blocked, likely due to supraphysiological levels of endogenous LPA. Further confirmation of these results was obtained with tests of spheroid formation and clonogenicity. As can be seen in Fig. 5, IOA-289 proved very effective in inhibiting the formation and growth of spheroids, in particular in the HLE and KKU-M213 cell lines, starting at the low concentration of 3 uM. In PANC-1 cells an inhibitory effect is, instead, observable only at a concentration of 12 uM. Furthermore, in all cell lines analysed, IOA-289 proved equally effective in inhibiting, in a dose-dependent manner, the ability of cells to form colonies starting from single cells (Fig. 6). In particular, in KKU-M213, HLE and HT-29 cells the formation of colonies with at least 50 cells was completely inhibited at the 12 uM concentration of IOA-289. Determination of the number of viable cells by colorimetry confirmed the inhibitory effect of IOA-289 on proliferation. Analysis of the cell cycle in all the cell lines examined showed that the effect of IOA-289 treatment on cell growth was to cause an arrest in S phase, particularly evident in KKU-M213, HLE and Panc-1 cells (Fig. 7).

### IOA-289 inhibits cell migration

**Fig. 8:**
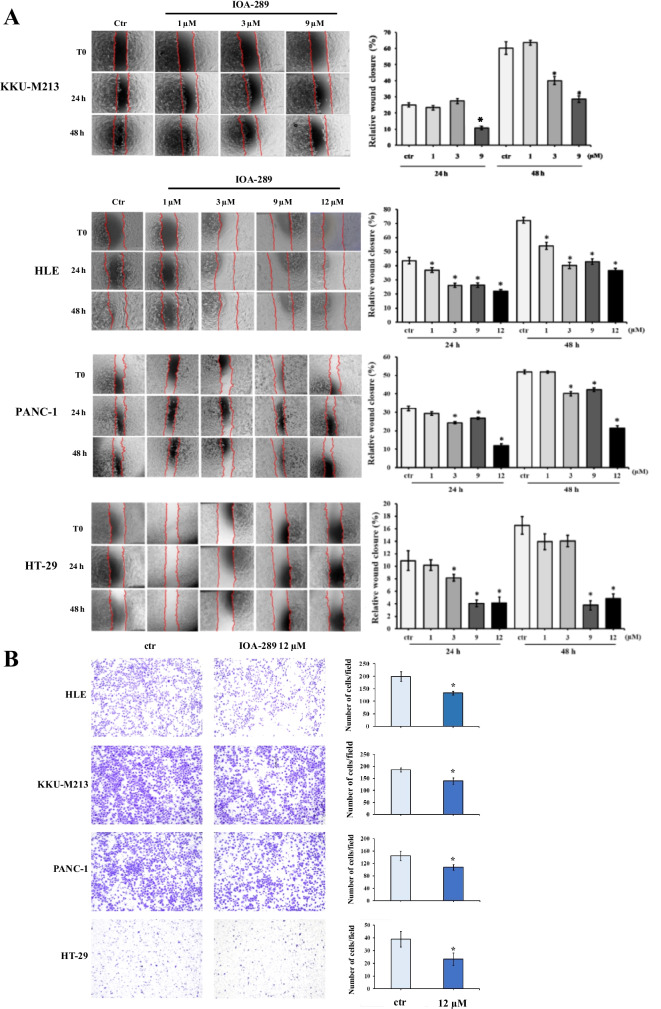
Effect of IOA-289 on cell migration.** A** Representative phase-contrast microscope images taken at 0, 24 and 48 h of a wound healing assay in cell cultures treated with IOA-289 in serum-free medium. Cell migration into the cell-free region (outlined) is inhibited in presence of the inhibitor, as compared to control. **B** Representative microscopic images of transwell migration assay of cells treated with 12 uM IOA-289 for 24 h, showing a decreased number of cells migrated through the membrane. *P < 0.05

Tumor progression is characterized by an enhanced cell motility and invasiveness. To expand our knowledge of the effects of IOA-289 on HLE, KKU-m213, HT-29 and Panc-1 cells, we assessed their migratory activity when treated with IOA-289, by both a wound healing and a transwell migration assay. Cells were seeded in a 12-well plate and, when a confluent monolayer was reached, an artificial gap was generated between the cells using a pipette tip. Scratch closure by migrating cells was observed after 48 h and 72 h. As reported in Fig. 8A, IOA-289 significantly (*p* < 0.05) reduced cell migration and wound closure of KKUM-213 cells treated with 9 $\mathrm{\mu M}$ IOA-289 (achieving an approximately 50% reduction in wound closure), compared to control, already after 24 h and 48 h of treatment. In HLE cells a concentration of 3 µM IOA-289 is already sufficient to cause a 40% inhibition of cell migration, whereas in PANC-1 cells a substantial reduction (60%) in migration was observed with the highest concentration of inhibitor, at 12 µ. In HT-29 cells 9 and 12 µM of IOA-289 significantly (*p* < 0.05) inhibited wound closure. Similarly, the transwell assay showed that IOA-289 treatment significantly inhibited the migratory capacity of all the tested cancer cell lines (Fig. 8B). In conclusion, IOA-289 can inhibit cancer cell migration.

### IOA-289 induces cell apoptosis

**Fig. 9:**
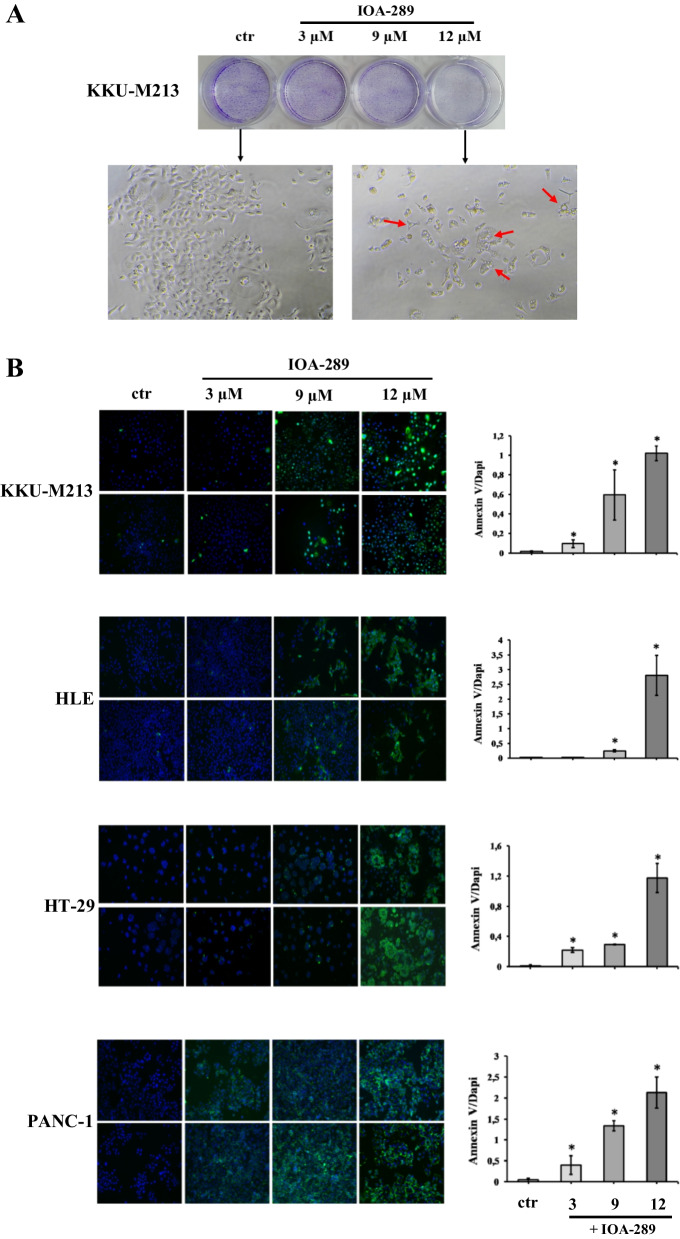
**A** Morphological changes of KKU-M213 cells observed under an inverted light microscope following treatment with 3, 9 and 12 µM IOA-289 for 72 h. Cell number can be observed to decrease with increasing concentrations of the inhibitor, as assessed by crystal violet staining. Moreover, the cells exhibit morphological changes and characteristics of apoptosis such as cell shrinkage and cytoplasmic vacuolization (red arrow). **B** IOA-289 induces apoptosis, in a dose-dependent manner, as observed by fluorescence microscopy after treatment with IOA-289 for 4 h and staining with Annexin V-FITC antibody. Annexin V positive cells are early apoptotic cells. Green fluorescence (Annexin V) was quantified using ImageJ software and correlated to DAPI blue fluorescence (nuclei). *P < 0.05

**Fig. 10:**
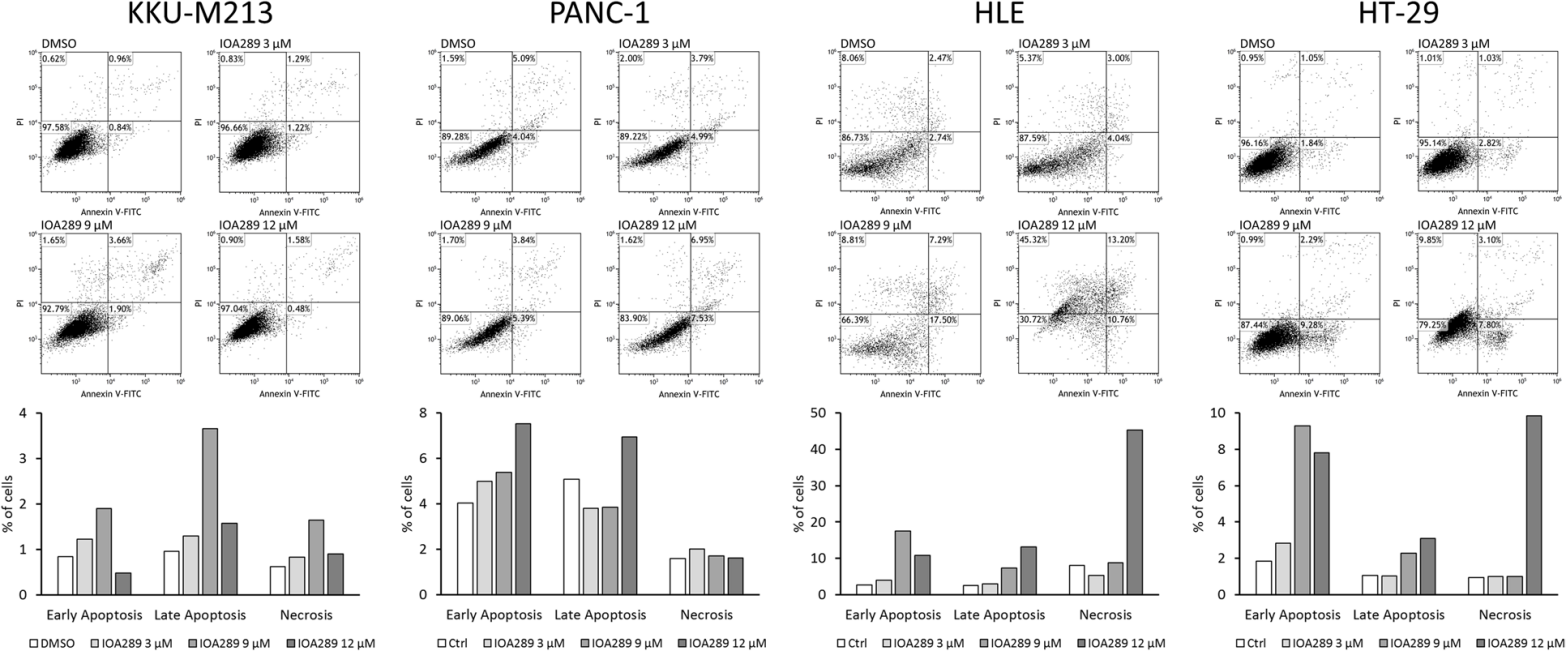
Analysis by flow cytometry of HLE, KKU-M213, PANC-1 and HT-29 induced-apoptosis and necrosis following treatment with IOA-289. Cells were cultured in absence or presence of the inhibitor (3, 9 and 12 µM concentrations) for 24 h, then they were labeled with Annexin V and propidium iodide prior to analysis with the flow cytometer

**Fig. 11:**
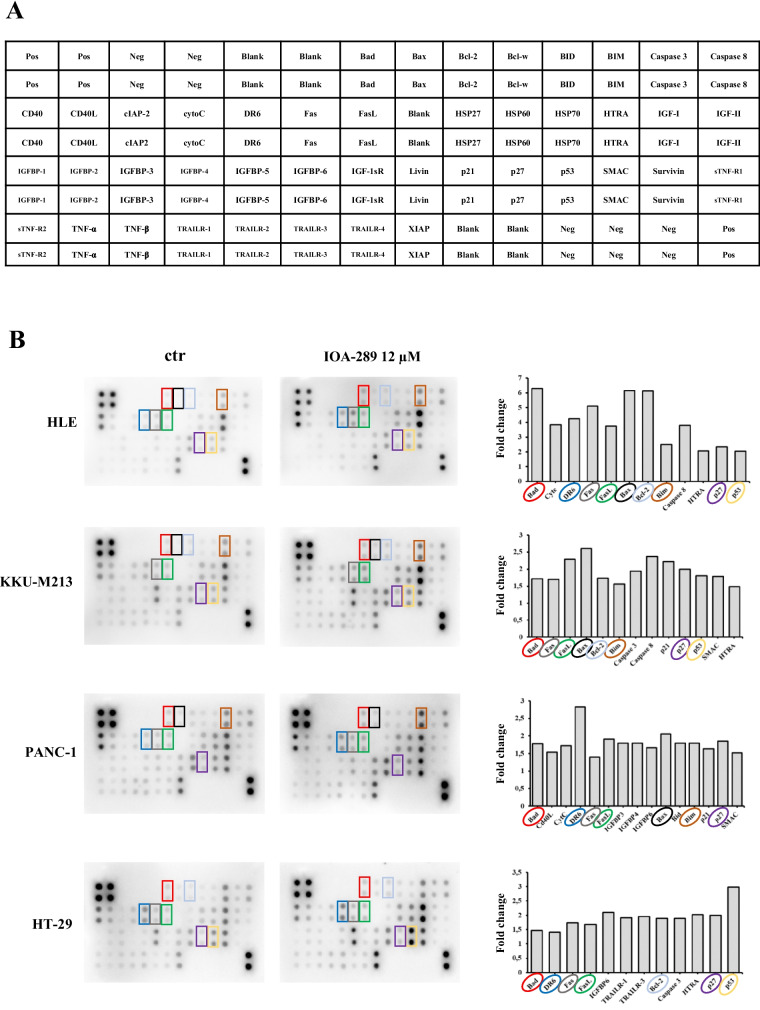
IOA-289 regulates cell apoptosis of HLE, KKU-M213, PANC-1 and HT-29 gastrointestinal cancer cell lines upregulating apoptosis-related proteins. A human apoptosis antibody array was used to identify up to 43 differentially expressed apoptosis-related proteins in untreated control cells compared to cells incubated with 12 uM IOA-289 for 24 h. Representative images were randomly selected from at least 3 independent experiments. In graph have been reported the differently expressed proteins in the cells treated with IOA-289, with the cut-off value set at a fold-change ≥ 1,5. Upregulated proteins in common between cell lines are highlighted with different colors

To further explore the activity of IOA-289 on gastrointestinal cancer cells, we investigated its effectiveness on inducing cell apoptosis. Following incubation with IOA-289, cells underwent morphological changes, featuring cell shrinkage and cytoplasmic vacuolization (Fig. 9A). An early event in apoptosis is the loss of plasma membrane asymmetry, resulting in the translocation of phosphatidylserine (PS) from the inner to the outer plasma membrane leaflet. Exposed PS on the cell surface can be used to measure apoptosis based on the binding with Annexin V. KKU-M213, HLE, HT-29, and PANC-1 cells were incubated with increasing concentrations of IOA-289 (3, 9 and 12 µM). After 4 h, cells were stained with a FITC-Annexin V antibody and fixed. In KKU-m213, HT-29 and Panc-1, IOA-289 significantly (*p* < 0.05) induced cell apoptosis at 3, 9 and 12 µM in a dose dependent manner, whereas in HLE cells the same effect was seen at 9 and 12 µM (Fig. 9B). To corroborate these results the same cancer cell lines were treated with the same concentrations of IOA-289 for 24 h and apoptosis/necrosis was analyzed by flow cytometry. A general increase of apoptosis and necrosis of KKU-M213, PANC-1, HLE, and HT-29 cells was observed, in a dose-dependent manner, consistently with the previously observed decrease of cell proliferation (Fig. 10). In addition, a human apoptosis antibody array was used to identify the differential expression of 43 apoptosis-related proteins in cells treated with 12 µM IOA-289 for 24 h as compared to untreated cells. As shown in Fig. 11, IOA-289 may promote cell apoptosis through the upregulation of pro-apoptotic proteins such as Bim, Caspase-3 and -8,, DR6, Fas, Fas-L, p53,SMAC and others.

## Discussion

LPA and ATX play a fundamental role in the progression of several tumors. Precisely because of this role, this axis may be an important therapeutic target. In recent years several ATX inhibitor molecules have been developed [19]. IOA-289 is an ATX inhibitor under development for the treatment of solid cancers, particularly those characterized by a high level of fibrosis [16].

In this study we characterized the biological effects of IOA-289 treatment on different tumor cell lines of the gastrointestinal tract: cholangiocarcinoma (KKU-M213, RBE), hepatocellular carcinoma (HLE, HLF), colorectal cancer (Caco2, HT-29) and pancreatic cancers (Panc-1, MIA PaCa-2). Consistently, IOA-289 hampered cell proliferation in all cell lines, in a dose-dependent manner, and with a stronger effect at later time points. Unexpectedly, the presence of FBS partially or completely inhibited the cytotoxic effects of IOA-289. We suspect that this may be due to supraphysiological concentrations of LPA in FBS, which consequently blunts the activity of IOA-289. In human fetal serum, LPA is known to be highly active and hence it is reasonable to assume that FBS contains similarly high concentretions [20]. Our findings are relevant for future in vitro studies with ATX inhibitors because FBS is commonly used to maintain viable cell cultures. Therefore, it is essential to be aware of the interference of FBS when assessing the anti-tumor activity of novel ATX inhibitors in cell cultures.

The mortality associated with gastrointestinal tumors is mainly related to the predisposition of these cancers to metastasize. For example, in animal models of breast cancer, IOA-289 has been shown to inhibit the dissemination of primary tumors [16]. Here, we add a new piece of evidence, namely that LPA inhibition is a key contributor to blocking metastasis. This is consistent with the role of LPA in stimulating tumor cell motility. For example, LPA stimulates the motility of DLD1 colon carcinoma cells [21] and regulates the migration of gastric cancer cells [22]. Through its receptor LPAR1, LPA induces the migration and expression of MMP9 in HCC cell lines [23]. The LPA, ATX and LPA receptors are widely expressed in different tumor types and at different degrees [24]. For example, LPAR5 is mainly expressed in stomach, colon and pancreatic cancer, while LPAR3 and LPAR4 are predominantly expressed in pancreatic cancer. This differential expression observed in patients may also be present in cell lines and hence explain the occasional difference in the response to IOA-289 blocking of LPA signaling.

We observed, both by wound healing assay and transwell migration assay, that inhibition of ATX by IOA-289 decreases the motility of tumor cell lines, in a dose-dependent manner. Several studies have demonstrated that ATX and LPA can protect cells from apoptosis and confer resistance to chemotherapeutic drugs, as has been observed, for example, in colon cancer cells [25, 26]. In our experiments, we were able to demonstrate that IOA-289 treatment determines the activation of the apoptotic pathway, as shown by exposure on the membrane of the phosphatidylserine residues, detectable by staining with Annexin V-FITC and by flow cytometry. Moreover, following treatment with IOA-289, several pro-apoptotic proteins are upregulated, such as BIM, Caspase-3 and -8, SMAC, Fas and HTRA, among others. In line with our observation in cancer cells, others authors have reported an LPA-dependent regulation in aortic smooth muscle cells [27]. Again, the concentration of FBS was key in blocking the effect of LPA-associated cell death.

It is well known that ATX plays a fundamental pathological role in pulmonary fibrosis. ATX, derived from the bronchial epithelium and alveolar macrophages catalyses the local production of LPA. In turn, LPA activates its cognate receptors and induces epithelial apoptosis, LPA and IL-8 secretion by epithelial cells and promotes inflammation. Moreover, LPA stimulate the α_v_β_6_-mediated TGFβ activation leading to the activation and trans-differentiation of pulmonary fibroblasts [28]. ATX inhibition was shown to attenuate pulmonary fibrosis [29], thus providing the rationale for therapeutic usage of ATX inhibitors, like IOA-289, in cancers characterised by a high degree of fibrosis, such as gastrointestinal cancers.

In summary, we show, for the first time that ATX inhibitors need to be evaluated with care in conditions that do not obscure the potential effect under more physiological conditions. Since more patients have tumors in a nutrient-depleted condition [30], the observations in the absence of FBS point to a potential direct tumor cell killing effect of ATX inhibition, which has been little appreciated until recently.

## Conclusions

In the fight against gastrointestinal tumors, the strategy of using drugs that target the ATX/LPA axis signaling pathway may offer a novel therapeutic option in association with common chemotherapy drugs, especially in the treatment of tumors characterized by a high level of fibrosis, such as pancreatic and liver cancer. In conclusion, our study demonstrates that, in this context, IOA-289 could play an important role. Plans to extend our in vitro observations to in vivo experimental models are underway to further support the clinical development of IOA-289, which has recently entered a Phase 1b study in patients with pancreatic cancer (NCT05586516).

## Data Availability

All data generated or analyzed during this study are included in this manuscript.

## References

[1] Solís KH (2021). The LPA3 Receptor: Regulation and Activation of Signaling Pathways. Int J Mol Sci..

[2] Geraldo LHM, Spohr TCLS, Amaral RFD, Fonseca ACCD, Garcia C, Mendes FA, Freitas C, dosSantos MF, Lima FRS (2021). Role of lysophosphatidic acid and its receptors in health and disease: novel therapeutic strategies. Signal Transduct Target Ther.

[3] Zhang X, Li M, Yin N, Zhang J (2021). The Expression Regulation and Biological Function of Autotaxin. Cells.

[4] Nakanaga K, Hama K, Aoki J (2010). Autotaxin-An LPA Producing Enzyme with Diverse Functions. J Biochem.

[5] van Meeteren LA, Moolenaar WH (2007). Regulation and biological activities of the autotaxin-LPA axis. Prog Lipid Res.

[6] Salgado-Polo F, Fish A, Matsoukas MT, Heidebrecht T, Keune WJ, Perrakis A (2018). Lysophosphatidic acid produced by autotaxin acts as an allosteric modulator of its catalytic efficiency. J Biol Chem..

[7] Hausmann J, Kamtekar S, Christodoulou E, Day JE, Wu T, Fulkerson Z, Albers HM, van Meeteren LA, Houben AJ, van Zeijl L, Jansen S, Andries M, Hall T (2011). Structural basis of substrate discrimination and integrin binding by autotaxin. Nat Struct Mol Biol.

[8] Fulkerson Z, Wu T, Sunkara M, Kooi CV, Morris AJ, Smyth SS (2011). Binding of autotaxin to integrins localizes lysophosphatidic acid production to platelets and mammalian cells. J Biol Chem.

[9] Yung YC, Stoddard NC, Chun J (2014). LPA receptor signaling: pharmacology, physiology, and pathophysiology. J Lipid Res.

[10] Barbayianni E, Kaffe E, Aidinis V, Kokotos G (2015). Autotaxin, a secreted lysophospholipase D, as a promising therapeutic target in chronic inflammation and cancer. Prog Lipid Res.

[11] Watanabe N, Ikeda H, Nakamura K, Ohkawa R, Kume Y, Aoki J, Hama K, Okudaira S, Tanaka M, Tomiya T (2007). Both plasma lysophosphatidic acid and serum autotaxin levels are increased in chronic hepatitis C. J Clin Gastroenterol.

[12] Cooper AB, Wu J, Lu D, Maluccio MA (2007). Is autotaxin (ENPP2) the link between hepatitis C and hepatocellular cancer?. J Gastrointest Surg.

[13] Aihua Xu, Khan MdAhsanul Kabir, Chen Fangzhi, Zhong Zhaohui, Chen Han-Chun, Song Yuanda (2016). Overexpression of autotaxin is associated with human renal cell carcinoma and bladder carcinoma and their progression. Med Oncol..

[14] Magkrioti C, Oikonomou N, Kaffe E, Mouratis MA, Xylourgidis N, Barbayianni I, Megadoukas P, Harokopos V, Valavanis C, Chun J (2018). The autotaxin-lysophosphatidic acid axis promotes lung carcinogenesis. Cancer Res..

[15] Banerjee S, Lee S, Norman DD, Tigyi GJ (2022). Designing Dual Inhibitors of Autotaxin-LPAR GPCR Axis. Molecules..

[16] Deken M, Niewola K, Matas-Rico E, Peyruchaud O, et al. Characterisation and translational development of IOA-289, a novel autotaxin inhibitor for the treatment of solid tumors. Immuno-Oncol Technol. 2023. 10.1016/j.iotech.2023.100384. 10.1016/j.iotech.2023.100384 PMC10205783 37234285

[17] Auciello FR, Bulusu V, Oon C, Tait-Mulder J, Berry M, Bhattacharyya S, Tumanov S, Allen-Petersen BL, Link J, Kendsersky ND, Vringer E, Schug M, Novo D, Hwang RF, Evans RM, Nixon C, Dorrell C, Morton JP, Norman JC, Sears RC, Kamphorst JJ, Sherman MH (2019). A Stromal Lysolipid-Autotaxin Signaling Axis Promotes Pancreatic Tumor Progression. Cancer Discov.

[18] Matas-Rico E, Frijlink E, van der Haar Àvila I, Menegakis A, van Zon M, Morris AJ, Koster J, Salgado-Polo F, de Kivit S, Lança T, Mazzocca A, Johnson Z, Haanen J, Schumacher TN, Perrakis A, Verbrugge I, van den Berg JH, Borst J, Moolenaar WH (2021). Autotaxin impedes anti-tumor immunity by suppressing chemotaxis and tumor infiltration of CD8^+^ T cells. Cell Rep..

[19] Matralis AN (2019). Development and therapeutic potential of autotaxin small molecule inhibitors: From bench to advanced clinical trials. Med Res Rev..

[20] Tokumura A, Kanaya Y, Miyake M, Yamano S, Irahara M, Fukuzawa K (2002). Increased production of bioactive lysophosphatidic acid by serum lysophospholipase D in human pregnancy. Biol Reprod.

[21] Shida D (2003). Lysophosphatidic acid (LPA) enhances the metastatic potential of human colon carcinoma DLD1 cells through LPA1. Cancer Res.

[22] Shida D (2004). Dual mode regulation of migration by lysophosphatidic acid in human gastric cancer cells. Exp Cell Res.

[23] Park SY, Jeong KJ, Panupinthu N, Yu S, Lee J, Han JW, Kim JM, Lee JS, Kang J, Park CG, Mills GB, Lee HY (2011). Lysophosphatidic acid augments human hepatocellular carcinoma cell invasion through LPA1 receptor and MMP-9 expression. Oncogene.

[24] Willier S (2013). Lysophosphatidic acid (LPA) signalling in cell migration and cancer invasion: a focussed review and analysis of LPA receptor gene expression on the basis of more than 1700 cancer microarrays. Biol Cell.

[25] Sun H, Ren J, Zhu Q, Kong FZ, Wu L, Pan BR (2009). Effects of lysophosphatidic acid on human colon cancer cells and its mechanisms of action. World J Gastroenterol.

[26] Deng W, Wang DA, Gosmanova E, Johnson LR, Tigyi G (2003). LPA protects intestinalepithelial cells from apoptosis by inhibiting the mitochondrial pathway. Am J Physiol Gastrointest Liver Physiol.

[27] Asai D, Kawano T, Murata M, Nakashima H, Toita R, Kang JH (2020). Effect of Fetal Bovine Serum Concentration on Lysophosphatidylcholine-mediated Proliferation and Apoptosis of Human Aortic Smooth Muscle Cells. J Oleo Sci.

[28] Ninou I, Magkrioti C, Aidinis V (2018). Autotaxin in Pathophysiology and Pulmonary Fibrosis. Front Med (Lausanne)..

[29] Oikonomou N, Mouratis MA, Tzouvelekis A, Kaffe E, Valavanis C, Vilaras G (2012). Pulmonary autotaxin expression contributes to the pathogenesis of pulmonary fibrosis. Am J Respir Cell Mol Biol.

[30] Kamphorst JJ (2015). Human pancreatic cancer tumors are nutrient poor and tumor cells actively scavenge extracellular protein. Cancer Res.

