# Correction: Autotaxin inhibitor IOA-289 reduces gastrointestinal cancer progression in preclinical models

**DOI:** 10.1186/s13046-023-02797-9

**Published:** 2023-08-18

**Authors:** Matteo Centonze, Giusy Di Conza, Michael Lahn, Isabel Fabregat, Francesco Dituri, Isabella Gigante, Grazia Serino, Rosanna Scialpi, Livianna Carrieri, Roberto Negro, Elena Pizzuto, Gianluigi Giannelli

**Affiliations:** 1 National Institute of Gastroenterology - IRCCS “Saverio de Bellis”, Via Turi 27, 70013 Castellana Grotte, Italy; 2 iOnctura SA, Avenue Secheron 15, 1202 Geneva, Switzerland; 3 TGF-β and Cancer Group, Oncobell Program, Bellvitge Biomedical Research Institute (IDIBELL) and CIBEREHD ? ISCIII, Barcelona, Spain

**Correction:** ***J Exp Clin Cancer Res*** **42,197 (2023)**

10.1186/s13046-023-02780-4

Following publication of the original article [1], an error was identified in the Discussion section. The updated sentence under Discussion is given below and the changes have been highlighted in **bold typeface**.

Unexpectedly, the presence of FBS partially or completely inhibited the cytotoxic effects of **IOA-289.**

The correction does not affect the overall conclusion of the article. The original article has been corrected.
